# Clinical Significance of Intermediate-Density Lipoprotein Cholesterol Determination as a Predictor for Coronary Heart Disease Risk in Middle-Aged Men

**DOI:** 10.3389/fcvm.2021.756057

**Published:** 2021-11-22

**Authors:** Hiroshi Yoshida, Kumie Ito, Daisuke Manita, Ryo Sato, Chika Hiraishi, Sadako Matsui, Yuji Hirowatari

**Affiliations:** ^1^Department of Laboratory Medicine, The Jikei University Kashiwa Hospital, Kashiwa, Japan; ^2^Internal Medicine of Metabolism and Nutrition, The Jikei University Graduate School of Medicine, Tokyo, Japan; ^3^Nihonbashi Sakura Clinic, Tokyo, Japan; ^4^Bioscience Division, TOSOH Corporation, Kanagawa, Japan; ^5^Department of Food and Nutrition, Faculty of Human Sciences and Design, Japan Women's University, Tokyo, Japan; ^6^Department of Health Science, Laboratory Science, Saitama Prefectural University, Saitama, Japan

**Keywords:** Framingham risk score, IDL-cholesterol, Non-HDL cholesterol, VLDL-cholesterol, Suita score

## Abstract

**Background:** Not only low-density lipoprotein (LDL) cholesterol but also non-high-density lipoprotein cholesterol (non-HDL-C), very low-density lipoprotein (VLDL) cholesterol (VLDL-C), and intermediate-density lipoprotein (IDL) cholesterol (IDL-C) are reported to be significant risk markers for coronary heart disease (CHD). We reported the relevance of IDL-C to Framingham risk score (F-score), but the present study addressed the relevance of IDL-C to Suita score (S-score), a risk score for coronary heart disease (CHD) developed for the Japanese individuals in addition to F-score.

**Methods:** The cholesterol levels of lipoproteins, including triglyceride (TG)-rich lipoproteins (IDL and VLDL), were measured by an anion exchange high-performance liquid chromatography (AEX-HPLC). This study enrolled 476 men, aged mean 51 years and free of CHD and stroke.

**Results:** Non-HDL-C, IDL-C, and VLDL-C significantly correlated with F-score and S-score. In the multiple stepwise regression analysis, IDL-C as well as body mass index (BMI) significantly correlated with both F-score and S-score in both the total subjects and the subjects without drug therapy. The multivariate logistic analysis with the model composed of BMI and IDL-C as the predictor variables demonstrated that 1 SD increase in IDL-C was an independent predictor for 10-year CHD risk >10% of F-score (*OR* 1.534, 95% CI 1.266–1.859, *p* < 0001) and that of S-score (*OR* 1.372, 95% CI 1.130–1.667, *p* = 0.0014) in the total subjects. Even in the subjects without the drug therapy, the increased IDL-C, as well as BMI, were significant predictors for 10-year CHD risk >10% of S-score as well as F-score.

**Conclusion:** These results suggest the significant relevance of the increased IDL-C for CHD risk scores in middle-aged men free of CHD and stroke. Further investigations are needed in women and elderly subjects.

## Introduction

A high level of serum low-density lipoprotein (LDL) cholesterol (LDL-C) is established as a primary risk factor for atherosclerotic cardiovascular disease (ASCVD), including coronary heart disease (CHD) (1–3). However, a residual ASCVD risk remains after LDL-C reduction under the target level by LDL-lowering therapy (1–6). In addition to LDL-C, total cholesterol (TC) minus high-density lipoprotein (HDL) cholesterol (HDL-C), namely non-HDL-C, is of importance as a risk marker for ASCVD (6–10). Non-HDL is composed of apolipoprotein B (ApoB)-containing lipoproteins, including triglyceride (TG)-rich lipoproteins [very low-density lipoprotein (VLDL), intermediate-density lipoprotein (IDL), and remnant lipoprotein]. Recently, a high level of non-HDL-C attracts attention because of its important significance and clinical usefulness in relation to the determination of ASCVD risk (6).

Not only LDL-C but also VLDL cholesterol (VLDL-C) and IDL cholesterol (IDL-C) are reported to be significant risk markers for CHD (7, 11, 12). The results from subjects aged ≥30 years and free of CHD at baseline in the Framingham Heart Study suggest that non-HDL-C and VLDL-C are stronger predictors of CHD risk than LDL-C regardless of the serum TG levels, indicating that VLDL-C may play a critical role in the development of CHD (7). The Copenhagen General Population Study reported that VLDL-C explained one-half myocardial infarction risk relevant to the cholesterol levels of ApoB-containing lipoproteins and indicated that IDL-C was a stronger predictor for myocardial infarction risk (11). In addition, VLDL-C explained a large fraction of excess myocardial infarction risk in obese individuals (12). We reported that IDL-C may contribute as a useful marker to CHD risk determination in the Japanese men free of CHD and stroke, indicating the significant association of increased IDL-C levels with high levels of Framingham risk score (F-score) (13).

As mentioned above, VLDL-C and IDL-C may be the significant markers for ASCVD risk. However, each method for the determination of VLDL-C and IDL-C is different among the previous studies. The measurement methods in the Framingham Heart Study and the Copenhagen General Population Study were ultracentrifugation (7) and an NMR spectroscopy platform (11, 12), respectively. On the other hand, our study used an anion exchange-high performance liquid chromatography (AEX-HPLC) method, convenient and inexpensive as compared with ultracentrifugation and NMR (13, 14). VLDL-C and IDL-C measured by the AEX-HPLC method are sufficiently correlated with those measured by an ultracentrifugation method (14–16). Meanwhile, the Suita score (S-score) has been established for predicting a 10-year probability of developing CHD, which is based on the findings of a large cohort study in Japan (17). The F-score overestimated the 10-year risk of CHD for the Japanese population as compared with the S-score (17).

Consequently, we investigated the relevance of cholesterol levels of TG-rich lipoproteins (VLDL-C and IDL-C), measured by the AEX-HPLC method, to CHD risk estimated by S-score and F-score in men free of CHD and stroke.

## Methods

The present cross-sectional study enrolled 476 middle-aged men who underwent annual medical checkup examination in Tobu Medical Center (Shizuoka, Japan), and who did not suffer from CHD, stroke, and any cancer according to the medical questionnaire. At entry, written informed consent was obtained from all the participants. The study protocol was approved by the institutional review board of Tobu Medical Center (approval no. 2010–01). In our previous study (13), 487 men were enrolled, but in the present study, 476 men were enrolled because of the assessment of SS scores targeted at individuals aged 35 years and over.

The dataset of our previous study (13) was used for the present study. The measurement methods for main laboratory data are given below. The cholesterol levels of five lipoprotein classes were measured by using AEX-HPLC as described previously (13–16). Briefly, the HPLC system was composed of non-porous polymer-based gel with diethylaminoethyl ligands as separation media and sodium perchlorate buffers as elution reagents. TC was calibrated using the Lipopropak calibrator (LT-S01A, TC 271.8 mg/dL) (Tosoh, Tokyo, Japan), the value of which was assigned according to the reference materials JCCRM223-36 (TC level 137.1, 171.4, and 207.3 mg/dL; ReCCs). The analysis conditions of AEX-HPLC were optimized with VLDL [density (d) < 1.006 g/ml, IDL (1.006 < d < 1.01 g/ml), LDL (1.019 < d < 1.06 g/ml), and HDL (d > 1.063 g/ml)], and the samples for the calibration procedure were separated by ultracentrifugation. Each lipoprotein cholesterol concentration measured by AEX-HPLC was correlated with those measured by the ultracentrifugation method, and the accuracy of these cholesterol levels was reported (14, 16). In the five lipoprotein classes determined by the AEX-HPLC method, the data of "other fraction,” include lipoprotein(a) in addition to chylomicron (14, 18).

Cholestest-CHO, Cholestest-HDL, Cholestest-LDL, Cholestest-TG (Sekisui Medical, Tokyo, Japan), GA08 (A and T Corp, Yokohama, Japan), and HLC-723G8 (Tosoh Corporation, Tokyo, Japan) were used to measure TC, HDL-C, LDL-C, TG, blood glucose, and hemoglobin (Hb) A1c, respectively. Non-HDL-C was calculated by subtracting HDL-cholesterol from TC. In addition, the estimated glomerular filtration rate (eGFR) was calculated using the following formula: 194 × creatinine −1.094 × age (years) −0.287 (19).

We determined F-score [National Cholesterol Education Program Adult Treatment Panel III (NCEP-ATPIII) version] levels of 476 subjects, incorporating data of age, sex, TC or LDL-C, and HDL-C concentrations, blood pressures, anti-hypertensive drug medication, smoking and diabetic status into the calculation (20, 21). The S-score levels also were calculated similarly, using data on age, sex, TC or LDL-C, HDL-C, SBP, DBP, smoking, diabetes, and eGFR (17, 22). A distinct difference between the two scores is that e-GFR is incorporated into S-score but not into F-score.

The data were presented as mean ± SD. Student's *t*-test or Mann–Whitney *U*-test was used to compare the variables between Group 1 (<6 points of F-score) and Group 2 (≥6 points of F-score) or between Group 3 (<41 points of S-score) and Group 4 (≥41 points of S-score). Namely, Groups 1 and 3 are regarded as being at a low-risk stage, and Groups 2 and 4 are regarded as being at a high-risk stage (10-year CHD risk more than 10%). Assuming an α level of 0.05, 80% power, and 0.3 effect size, the required number of patients for each group to observe a difference in IDL-C was determined ≥154 in Group 1 and ≥230 in Group 2. The correlations were estimated by Spearman's rank test. A multiple stepwise regression analysis was performed to assess the independent relationship of the variables, body mass index (BMI), and cholesterol levels of IDL, VLDL, and the other fraction [chylomicron and lipoprotein(a)] (14, 18). The TC or LDL-C and HDL-C concentrations were incorporated into the calculation of F-score and S-score. Therefore, TC, LDL-C, and non-HDL-C were not applied to the explanatory factors of multivariate analysis. TG was considered as one of the explanatory factors for F-score and S-score, but TG also was not applied to the explanatory factors of multivariate analysis because of natural collinearity between TG and TG-rich lipoprotein cholesterol (VLDL-C and IDL-C).

In addition, the univariate and multivariate logistic regression analyses were performed to analyze the relationship between the nominal variables (high-risk stage of 10-year CHD risk at F-score points with ≥6 or S-score points with ≥41) and continuous variables (1 SD increase in BMI and cholesterol levels of TG-rich lipoproteins), with the results expressed as odds ratios (*OR*) and 95% *CI*s, and the predictive values of BMI and TG-rich lipoprotein cholesterol for the high-risk stage were investigated. The *P* values < 0.05 were considered significant. The statistical analyses were performed using STATFLEX software (version 7.0, Artech, Osaka, Japan).

## Results

The clinical characteristics, biochemical data, F-score, and S-score are shown in Table 1. The predicted 10-year CHD risk values (7–8%) calculated by the F-score (4.5 points) were higher than those (about 2%) estimated by the S-score (42.1 points) as reported previously (17). In Table 1, Groups 1 and 2 show data of subjects with F-score < 6 points and F-score ≥ 6 points, respectively. The percentages of subjects with hypertension and dyslipidemia were higher in Group 2 than in Group 1. Groups 3 and 4 show data of subjects with S-score <41 points and S-score ≥41 points, respectively. The patients with hypertension but not with dyslipidemia were more common in Group 4 than in Group 3. However, the prevalence of patients with diabetes was comparable both between Groups 1 and 2 and between Groups 3 and 4.

**Table 1:** Clinical characteristics, biochemical data, Framingham risk score and Suita score of the study subjects.

	**Total**	**Group 1**	**Group 2**	***P*-value**	**Group 3**	**Group 4**	***P*-value**
		**FRS < 6 points**	**FRS > 6 points**	**Group 1 vs**.	**SS < 41 points**	**SS > 41 points**	**Group 3 vs**.
	**(*n* = 476)**	**(*n* = 294)**	**(*n* = 182)**	**Group 2**	**(*n* = 202)**	**(*n* = 274)**	**Group 4**
Framingham risk score; FRS (total point)	4.5 ± 2.9	2.7 ± 2.1	7.5 ± 1.5	<0.0001	2.1 ± 2.1	6.4 ± 2.0	<0.0001
Suita score; SS (total point)	42.1 ± 9.8	37 ± 7	51 ± 6	<0.0001	33 ± 5	49 ± 6	<0.0001
**Basic data**							
Age (years)	51 ± 8	49 ± 7	56 ± 7	<0.0001	46 ± 6	55 ± 6	<0.0001
Body mass index (kg/cm^2^)	24.2 ± 3.2	23.7 ± 3.0	24.9 ± 3.4	<0.0001	23.5 ± 2.9	24.7 ± 3.3	<0.0001
Syematic blood pressure (mmHg)	122 ± 15	118 ± 13	129 ± 16	<0.0001	115 ± 13	128 ± 14	<0.0001
Diastonic blood pressure (mmHg)	77 ± 10	75 ± 10	81 ± 10	<0.0001	73 ± 10	80 ± 9	<0.0001
Smoker, *n* (%)	194 (41)	90 (31)	104 (57)	<0.0001	68 (34)	126 (46)	<0.01
Fasting blood glucose (mmol/L)	5.84 ± 0.99	5.64 ± 0.71	6.18 ± 1.25	<0.0001	5.55 ± 0.64	6.06 ± 1.13	<0.0001
Glycated hemoglobin A1c (%)	5.9 ± 0.6	5.8 ± 0.5	6.1 ± 0.8	<0.0001	5.7 ± 0.5	6.0 ± 0.7	<0.0001
Estimated GFR (mL/min/1.73 m^2^)	79.7 ± 13.9	80.9 ± 13.7	77.7 ±14.1	<0.05	82.3 ± 12.9	77.8 ± 14.4	<0.0005
**Lipid data**							
Total cholesterol (mmol/L)	5.29 ± 0.84	5.15 ± 0.82	5.52 ± 0.83	<0.0001	5.10 ± 0.80	5.44 ± 0.84	<0.0001
HDL cholesterol (mmol/L)	1.47 ± 0.35	1.55 ± 0.36	1.34 ± 0.29	<0.0001	1.53 ± 0.33	1.43 ± 0.36	<0.005
LDL cholesterol (mmol/L)	3.31 ± 0.80	3.13 ± 0.76	3.60 ± 0.78	<0.0001	3.11 ± 0.74	3.46 ± 0.81	<0.0001
Non-HDL cholesterol (mmol/L)	3.82 ± 0.89	3.60 ± 0.84	4.18 ± 0.83	<0.0001	3.60 ± 0.84	4.01 ± 0.87	<0.0001
Triglyceride (mmol/L)	1.56 ± 1.01	1.37 ± 0.85	1.85 ± 1.17	<0.0001	1.35 ± 0.83	1.71 ± 1.10	<0.0001
**Lipoprotein data by anion-exchange liquid chromatography**							
HDL cholesterol (mmol/L)	1.41 ± 0.37	1.49 ± 0.38	1.28 ± 0.31	<0.0001	1.47 ± 0.35	1.37 ± 0.38	<0.005
LDL cholesterol (mmol/L)	3.55 ± 0.87	3.37 ± 0.81	3.83 ± 0.88	<0.0001	3.36 ± 0.81	3.69 ± 0.88	<0.0001
IDL cholesterol (mmol/L)	0.203 ± 0.081	0.190 ± 0.081	0.224 ± 0.078	<0.0001	0.189 ± 0.081	0.213 ± 0.080	<0.005
VLDL cholesterol (mmol/L)	0.49 ± 0.39	0.42 ± 0.33	0.59 ± 0.46	<0.0001	0.42 ± 0.32	0.53 ± 0.43	<0.005
Other cholesterol (mmol/L)	0.104 ± 0.068	0.108 ± 0.077	0.099 ± 0.051	NS	0.112 ± 0.083	0.099 ± 0.055	NS
**Therapy for diseases**							
Hypertension, *n* (%)	83 (17)	43 (15)	40 (22)	<0.05	18 (8.9)	65 (24)	<0.0001
Dyslipidemia, *n* (%)	55 (12)	27 (9.2)	28 (15)	<0.05	19 (9.4)	36 (13)	NS
Diabeties mellitus, *n* (%)	32 (6.7)	15 (5.1)	17 (9.3)	NS	9 (4.5)	23 (8.4)	NS

*Data are expressed as means ± SD. NS means “not significant”*.

Both the F-score and S-score were calculated by age, sex-difference, TC or LDL-C, HDL-C, blood pressures, and status of smoking and glycemic control, and S-score also incorporated eGFR into the calculation. In the lipid data, LDL-C and HDL-C were excluded from the investigation because they were used in the calculation of risk scores. TC, TG, and non-HDL-C significantly correlated with the levels of F-score and S-score in the 476 men (Table 2). In TG-rich lipoproteins of non-HDL, IDL-C and VLDL-C significantly correlated with F-score and S-score. However, the individuals treated with drug therapy for hypertension, dyslipidemia, and diabetes were included in the 476 men (Table 1). Then, a part of the study subjects (*n* = 341) without the drug treatment was further investigated. Any drug users for dyslipidemia, diabetes, and hypertension were excluded from the sub-study subjects. The medication information was acquired from the annual medical checkup records. Table 2 shows the similar correlations of lipid levels to F-score and S-score in the 341 subjects as in the total subjects (*n* = 476). Furthermore, BMI significantly correlated with F-score and S-score both in the subjects without the drug therapy and in the total subjects (Table 2).

**Table 2:** Simple correlations of body mass index and serum lipids to Framingham risk score and Suita score.

	**Framingham risk score**			**Suita score**		
	**Rank correlation coefficient**	***t*-value**	***P*-value**	**Rank correlation coefficient**	***t*-value**	***P*-value**
**Total subjects (*****n*** **= 476)**						
**Basic data**						
Body mass index (kg/m^2^)	0.218	4.867	<0.0001	0.194	4.295	<0.0001
**Lipid data**						
Total cholesterol (mmol/L)	0.297	6.778	<0.0001	0.234	5.239	<0.0001
Non-HDL cholesterol (mmol/L)	0.417	9.978	<0.0001	0.295	6.727	<0.0001
Triglyceride (mmol/L)	0.338	7.821	<0.0001	0.255	5.747	<0.0001
**Lipoprotein data by anion-exchange liquid chromatography**						
IDL cholesterol (mmol/L)	0.313	7.181	<0.0001	0.240	5.391	<0.0001
VLDL cholesterol (mmol/L)	0.288	6.557	<0.0001	0.190	4.220	<0.0001
Other cholesterol (mmol/L)	0.008	0.170	NS	−0.035	−0.760	NS
**Subjects without drug therapy (*****n*** **= 341)**						
**Basic data**						
Body mass index (kg/m^2^)	0.211	3.978	<0.0001	0.183	3.429	<0.001
**Lipid data**						
Total cholesterol (mmol/L)	0.344	6.744	<0.0001	0.313	6.071	<0.0001
Non-HDL cholesterol (mmol/L)	0.457	9.467	<0.0001	0.362	7.140	<0.0001
Triglyceride (mmol/L)	0.366	7.247	<0.0001	0.272	5.212	<0.0001
**Lipoprotein data by anion-exchange liquid chromatography**						
IDL cholesterol (mmol/L)	0.313	6.069	<0.0001	0.241	4.577	<0.0001
VLDL cholesterol (mmol/L)	0.313	6.070	<0.0001	0.211	3.966	<0.0001
Other cholesterol (mmol/L)	−0.008	−0.147	NS	−0.054	−0.990	NS

*NS means “not significant”. Other cholesterol means cholesterol of chylomicron and lipoprotein(a)*.

Subsequently, a multiple stepwise regression analysis was performed to test the independent relationships of IDL-C and VLDL-C with F-score and S-score (Table 3). At first, BMI independently correlated with F-score and S-score levels both in the total subjects and in the subjects without the drug therapy. In the total subjects, IDL-C independently correlated with both the F-score and S-score levels, but the independent correlation of VLDL-C was found only in F-score. In the subjects without drug therapy, only IDL-C independently correlated with the F-score and S-score levels.

**Table 3:** Multiple stepwise regression of body mass index and cholesterol levels of triglyceride-rich lipoproteins to Framingham risk score and Suita score.

	**Framingham risk score**			**Suita score**		
	**Partial correlation coefficient**	***t*-value**	***P*-value**	**Partial correlation coefficient**	***t*-value**	***P*-value**
**Total subjects (*****n*** **= 476)**						
**Basic data**						
Body mass index (kg/m^2^)	0.173	3.930	0.0001	0.154	3.410	0.0007
**Lipoprotein data by anion-exchange liquid chromatography**						
IDL cholesterol (mmol/L)	0.217	4.407	<0.0001	0.187	3.698	0.0002
VLDL cholesterol (mmol/L)	0.111	2.204	0.0280	0.044	0.851	0.3950
Other cholesterol (mmol/L)	−0.074	1.715	0.0870	−0.071	1.603	0.1096
**Subjects without drug therapy (*****n*** **= 341)**						
**Basic data**						
Body mass index (kg/m^2^)	0.160	2.959	0.0033	0.157	2.817	0.0051
**Lipoprotein data by anion-exchange liquid chromatography**						
IDL cholesterol (mmol/L)	0.192	3.228	0.0014	0.170	2.783	0.0057
VLDL cholesterol (mmol/L)	0.118	1.885	0.0604	0.021	0.331	0.7411
Other cholesterol (mmol/L)	−0.085	1.664	0.0972	−0.094	1.782	0.0757

*Other cholesterol means cholesterol of chylomicron and lipoprotein(a)*.

**Figure 1:**
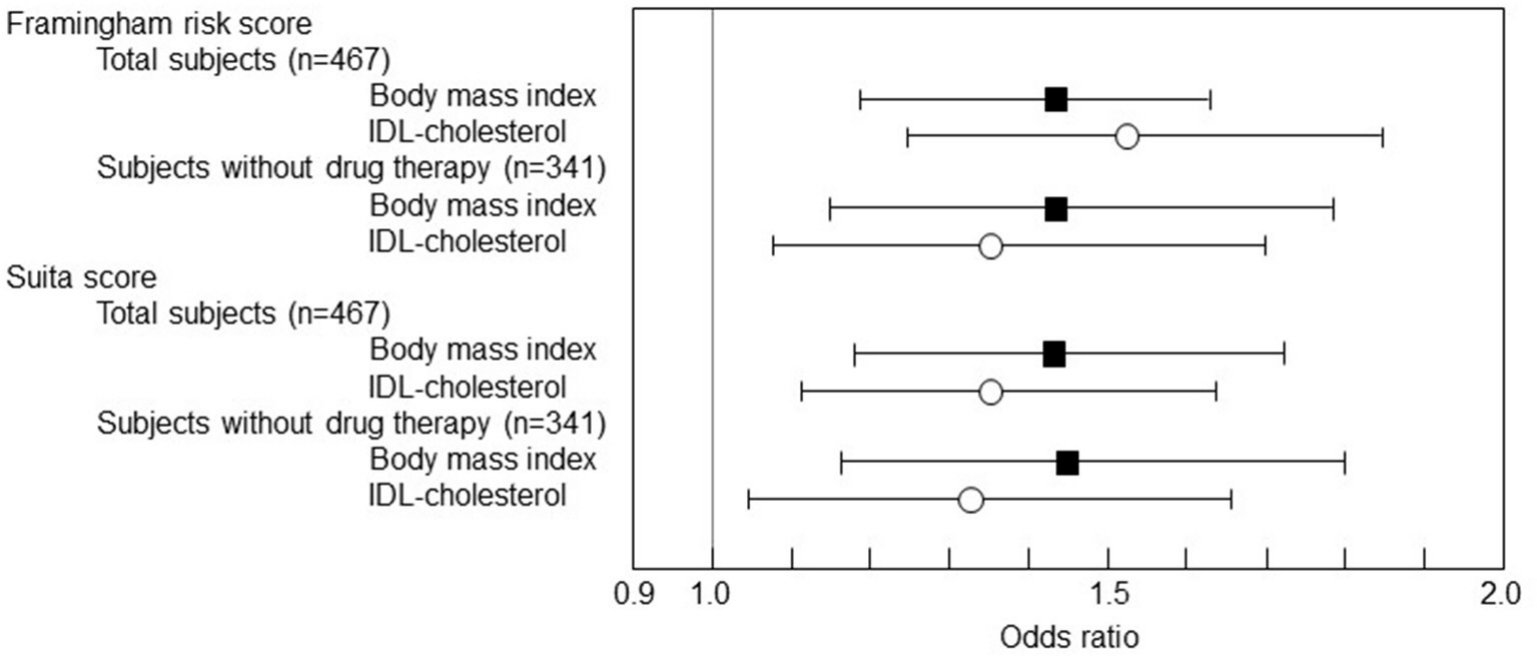
A univariate logistic regression analysis of body mass index (BMI) and cholesterol levels of intermediate-density lipoprotein (IDL) to Framingham risk score (F-score) and Suita score (S-score). The logistic regression results were shown as odds ratios (*OR*s) and 95% *CIs*. The 1 SD increase in BMI was significantly associated with F-score 10-year CHD risk> 10% (*OR* 1.430, 95% *CI* 1.187–1.722, *p* = 0.002 in total subjects and *OR* 1.431, 95% *CI* 1.146–1.787, *p* < 0.0001 in the subjects without drug therapy). The 1 SD increase in BMI was also associated with S-score 10-year CHD risk >10% (*OR* 1.420, 95% *CI* 1.175–1.717, *p* = 0.0003 in total subjects and *OR* 1.445, 95% *CI* 1.158–1.804, *p* = 0.0011 in the subjects without drug therapy). Besides, the 1 SD increase in IDL-C was associated with F-score 10-year CHD risk >10% (*OR* 1.520, 95% *CI* 1.248–1.850, *p* < 0.0001 in total subjects and *OR* 1.350, 95% *CI* 1.070–1.703, *p* = 0.0114 in the subjects without drug therapy) and also associated with the S-score 10-year CHD risk> 10% (*OR* 1.348, 95% *CI* 1.107–1.642, *p* = 0.003 in total subjects and *OR* 1.319, 95% *CI* 1.047–1.662, *p* = 0.019 in the subjects without drug therapy).

These results as above show that the high levels of IDL-C would be a potent marker for the F-score and S-score high-risk stage (10-year CHD risk >10%) as well as BMI. Then, the predictive values of BMI and IDL-C for 10-year CHD risk >10% were investigated by the univariate logistic regression analysis (Figure 1). The 1 SD increase in BMI was significantly associated with the F-score 10-year CHD risk >10% (*OR* 1.430 in total subjects and *OR* 1.431 in the subjects without drug therapy). In addition, the 1 SD increase in BMI was significantly associated with the S-score 10-year CHD risk >10% (*OR* 1.420 in total subjects and *OR* 1.445 in the subjects without drug therapy). Besides, the 1 SD increase in IDL-C was significantly associated with the F-score 10-year CHD risk >10% (*OR* 1.520 in total subjects and *OR* 1.350 in the subjects without drug therapy) and with S-score 10-year CHD risk >10% (*OR* 1.348 in total subjects and *OR* 1.319 in the subjects without drug therapy).

**Figure 2:**
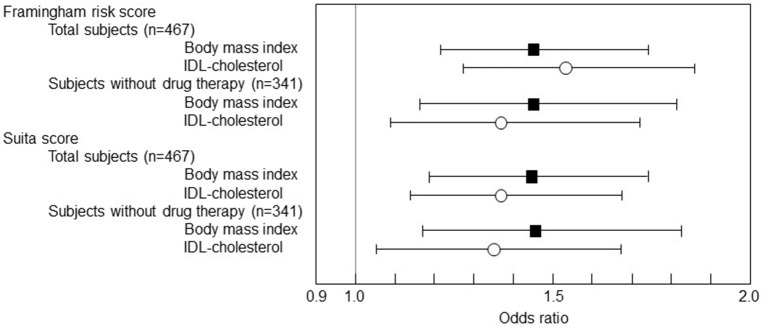
The multivariate logistic regression analysis of BMI and cholesterol levels of IDL to F-score and S-score. The logistic regression results were shown as *ORs* and 95% *CI*s. The 1 SD increase in BMI was significantly associated with F-score 10-year CHD risk > 10% (*OR* 1.448, 95% *CI* 1.206–1.738, *p* = 0.0001 in total subjects and *OR* 1.450, 95% *CI* 1.163-1.808, *p* < 0.0001 in the subjects without drug therapy). The 1 SD increase in BMI was also associated with S-score 10-year CHD risk > 10% (*OR* 1.441, 95% *CI* 1.194–1.739, *p* = 0.0001 in total subjects and *OR* 1.462, 95% *CI* 1.173–1.823, *p* = 0.0007 in the subjects without drug therapy). Besides, the 1 SD increase in IDL-C was associated with F-score 10-year CHD risk > 10% (*OR* 1.534, 95% *CI* 1.266–1.859, *p* < 0.0001 in total subjects and *OR* 1.368, 95% *CI* 1.091–1.716, *p* = 0.0066 in the subjects without drug therapy) and also associated with S-score 10-year CHD risk > 10% (*OR* 1.372, 95% *CI* 1.130–1.667, *p* = 0.0014 in total subjects and *OR* 1.337, 95% *CI* 1.067–1.675, *p* = 0.0116 in the subjects without drug therapy).

Subsequently, a multivariate logistic regression analysis was performed with the 1 SD increase in BMI and IDL-C as the predictor variables in the multivariate analysis model (Figure 2). IDL-C and BMI were independent predictive markers for the F-score and S-score high-risk stage of 10-year CHD risk >10% both in the total subjects and in the subjects without the drug therapy.

## Discussion

The present study demonstrates that the increased levels of IDL-C among the TG-rich lipoproteins of non-HDL significantly correlated with the levels of F-score and S-score independently of BMI, and also showed that the increased IDL-C would be a predictor for F-score and S-score 10-year CHD risk more than 10% in men free of CHD and stroke.

The previous papers from Framingham Heart Study and Copenhagen General Population Study demonstrate that the elevated levels of VLDL-C provide a certain contribution to ASCVD risk among the cholesterol levels of non-HDL, namely ApoB-containing lipoproteins (7, 11, 12). In our cross-sectional study with 476 individuals without CHD and stroke, however, we found the significant relevance of IDL-C rather than VLDL-C to CHD risk scores (FRS and SS). In the Copenhagen General Population Study, the multivariate-adjusted hazard ratios (*HR*s) for myocardial infarction for a 1-mmol/L (39 mg/dl) higher cholesterol content were 5.38 (95% *CI*: 3.73–7.75) for IDL, 2.07 (95% *CI*: 1.81–2.36) for VLDL, 1.86 (95% *CI*: 1.62–2.14) for LDL, and 1.49 (95% *CI*: 1.39–1.60) for non-HDL, presumably indicating the remarkable relevance of increased IDL-C to myocardial infarction risk (11). This attributable risk of IDL-C to myocardial infarction is presumably similar in effect to the IDL-C association with F-score and S-score in the present study. The similar messages from these previous cohort studies (Framingham Heart Study and Copenhagen General Population Study) show the significant contribution of elevated VLDL-C levels to CHD risk, but the present study suggested that the increased IDL-C rather than VLDL-C among the TG-rich lipoproteins significantly correlated with the levels of F-score and S-score. This discrepancy might be attributable in part to the differences in the methods for the determination of VLDL and IDL between the two studies and the present study. The Monitored Atherosclerosis Regression Study (MARS, *n* = 180), using data of IDL and VLDL measured ultracentrifugally, demonstrated that IDL but not VLDL or LDL was associated with the progression of carotid artery intima-media thickness, suggesting evidence for the atherogenicity of IDL independent of the levels of LDL and VLDL (23). However, which is a better predictor of CHD risk between VLDL-C and IDL-C remains inconclusive and it needs further large-scaled investigations.

Nishimura et al. reported that F-score might overestimate the CHD incidence in the Japanese general population, while S-score could improve the estimation power for CHD risk in the Japanese individuals (17). However, another study reported that the discrimination of S-score for estimating CHD was slightly better compared with F-score in whole individuals, but that the performance was comparable when the study subjects were divided into men and women (24). The reason for more accurately predicting CHD events by S-score than F-score in the Japanese individuals might be due to the incorporation of CKD factor into S-score calculation (17, 24). The incidence of myocardial infarction in the Japanese patients with hemodialysis with no history of ASCVD was independently associated with high non-HDL-C and low HDL-C, indicating that the elevated non-HDL-C predicts ASCVD events in the patients with hemodialysis (25–27). Especially, the increased IDL-C and decreased HDL-cholesterol levels in the patients with hemodialysis persisted even at very-low levels of serum lipids (27–30). In the patients with diabetes, VLDL-C was elevated but did not differ among the stages of diabetic nephropathy, whereas IDL-C was increasingly higher as the disease stage was advanced (27, 28). The previous studies with the AEX-HPLC method also showed increased levels of IDL-C and VLDL-C in the patients undergoing hemodialysis or continuous ambulatory peritoneal dialysis (CAPD) as compared with the healthy subjects (29, 30). In addition, elevated IDL-C levels in the patients with CAPD were found regardless of CAPD duration (30). The increased IDL-C would be a significant biomarker for CHD risk in individuals with kidney dysfunction.

Then, we previously reported that the cholesterol levels of IDL in TG-rich lipoproteins were significantly correlated with the F-score independently of BMI, regardless of the medications for dyslipidemia, diabetes, and hypertension although the multivariate logistic regression analysis was not performed (13). In the present study, IDL-C was significantly correlated not only with the F-score but also with the S-score, incorporating CKD in the CHD risk score calculation. Tatami et al. reported that the increased IDL-C was associated with the severity of coronary artery disease, estimated by the coronary lesion scores determined by coronary angiographic data, indicating the contribution of IDL to the development of CAD (31). Consequently, an increase in IDL-C among the cholesterol levels of non-HDL lipoproteins may be considered a more significant biomarker for ASCVD.

## Limitations

The present study has several limitations that need to be mentioned. One of the limitations is that this research was a cross-sectional study, which provides no evidence of a causal relationship between the IDL-C and ASCVD. Second, the interpretation of study results is limited to Japanese middle-aged men, and the extrapolation to other populations, such as women and elderly subjects should be validated by further studies. Third, because the methods to measure IDL-C and VLDL-C were different between the present study and the previous two studies, the direct comparison about the clinical significance of IDL-C and VLDL-C as an ASCVD risk biomarker could not be discussed. Fourth, IDL is considered a transient intermediate in the delipidation cascade from VLDL to LDL but also is known to be increased in the patients with high risk for CHD, including diabetes and kidney dysfunction. The normalization of ApoB-containing lipoprotein cholesterol levels by ApoB concentrations could adjust the individual status of LDL receptor activity and TG-rich lipoprotein metabolism, but unfortunately, the present study did not measure the ApoB concentrations.

## Conclusion

In conclusion, these results for the first time suggest the significant relevance of increased IDL-C for CHD risk score estimated by S-score as well as F-score in middle-aged men free of CHD and stroke. Admittedly, non-HDL-cholesterol is simple and inexpensive as a potential marker of ASCVD risk but is just the aggregated cholesterol amount of ApoB-containing lipoproteins. Therefore, when non-HDL-cholesterol is high, IDL-cholesterol is considered a CHD risk biomarker to be measured in middle-aged men.

## Data Availability Statement

The raw data supporting the conclusions of this article will be made available by the authors, without undue reservation.

## Ethics Statement

The studies involving human participants were reviewed and approved by the Institutional Review Board of Tobu Medical Center. The patients/participants provided their written informed consent to participate in this study.

## Author Contributions

HY was mainly responsible for writing this paper. YH and SM mainly handled the statistical analyses. KI mainly took charge of collecting the samples and data. All authors confirmed they have contributed to the intellectual content of this paper, have discussed the data of this study, and have read the manuscript.

## Funding

This study was supported by the Grant-in-Aid for Scientific Research (Number 17K09560) from the Japan Ministry of Education, Culture, Sports, Science, and Technology, the Jikei University Research Fund from the Jikei University School of Medicine (HY), Saitama Prefectural University Research Fund (YH), and Tosoh Corporation (DM and YH). We appreciate Norio Tada MD, PhD, visiting professor of the Jikei University, for his helpful comments.

## Conflict of Interest

This study received funding from the Jikei University Research Fund from the Jikei University School of Medicine (HY), Saitama Prefectural University Research Fund (YH), and Tosoh Corporation (DM and YH). The funder was not involved in the study design, collection, analysis, interpretation of data, the writing of this article or the decision to submit it for publication. HY received honoraria for speaking activities from Bayer, Denka, Kowa, Takeda, and Tosoh, but the above competing interests were not associated with the present study. DM is an employee of Tosoh. The remaining authors declare that the research was conducted in the absence of any commercial or financial relationships that could be construed as a potential conflict of interest.

## Publisher's Note

All claims expressed in this article are solely those of the authors and do not necessarily represent those of their affiliated organizations, or those of the publisher, the editors and the reviewers. Any product that may be evaluated in this article, or claim that may be made by its manufacturer, is not guaranteed or endorsed by the publisher.
